# Madder (*Rubia cordifolia L.*) Alleviates Myocardial Ischemia-Reperfusion Injury by Protecting Endothelial Cells from Apoptosis and Inflammation

**DOI:** 10.1155/2023/5015039

**Published:** 2023-02-23

**Authors:** Jinwei Gao, Zheng Wang, Zhangzhang Ye

**Affiliations:** Department of Cardiovascular Medicine, Qionghai People's Hospital, Qionghai, 571400 Hainan, China

## Abstract

**Objective:**

Ischemia-reperfusion injury often occurs in organ transplantation, coronary heart disease, ischemic heart disease, and other diseases, which greatly reduces clinical efficacy. This study examined the effectiveness of madder as a medicine to treat ischemia-reperfusion injury.

**Methods:**

The efficacy of madder was evaluated by measuring myocardial infarction size, coronary outflow volume, myocardial contraction rate, activation of inflammatory factors, autophagy factors, apoptosis factors, and related pathway genes in mice.

**Results:**

The results indicated that treatment with madder can effectively reduce the area of myocardial infarction and restore arterial blood flow velocity and myocardial contractility in mice. Additionally, madder treatment inhibited the expression of inflammatory factors, autophagy factors, and apoptosis factors in mice and reduced the degree of myocardial cell injury. Studies have also shown that madder treatment can alleviate myocardial ischemia-reperfusion injury in mice and inhibit the occurrence of inflammatory response by inhibiting the activity of the NF-*κ*B pathway.

**Conclusion:**

The results showed that madder was effective against ischemia-reperfusion injury, thus showing potential as a clinical drug for treating ischemia-reperfusion injury.

## 1. Introduction

The progress in shock treatment and the application of arterial bypass grafting, thrombolytic therapy, cardiopulmonary cerebral resuscitation, limb replantation, and organ transplantation have improved recovery after reperfusion of ischemic organs. Although some organ functions recover after reperfusion, sometimes, it can aggravate tissue damage. Reperfusion can lead to tissue cell injury or even necrosis, which is called ischemia-reperfusion (I/R) injury [1]. Ischemia-reperfusion can cause many diseases such as coronary heart disease and ischemic heart disease. Ischemic heart disease has high mortality. Restoring coronary blood flow is an important step in treating ischemic heart disease, but I/R injury seriously affects the effectiveness of treatment. Effective prevention and treatment of I/R injury are the keys to improve the quality of life of patients with ischemic heart disease [2]. Rupture of coronary atherosclerotic plaque can lead to thrombosis in coronary arteries, blockage of blood vessels, and myocardial infarction in severe cases [3]. Thrombolytic therapy or percutaneous coronary artery therapy is an effective treatment. However, the process of restoring blood supply after myocardial ischemia can cause reinjury, aggravate the injury, and seriously affect clinical efficacy. Studies have shown that myocardial cells are more likely to be damaged during ischemia, whereas endothelial cells are more likely to be damaged during reperfusion [4]. Prolonged cell damage induced by ischemia-reperfusion can lead to apoptosis, autophagy, and necrosis [5–7].

*Rubia cordifolia* L. (madder) is a plant with a long history of medicinal uses from which a red dye, also known as blood madder, blood see sorrow, is produced in Anhui, Jiangsu, Henan, Shaanxi, Shandong, and other places. Its medicinal part is the dried root [8]. The main medicinal functions of *R. cordifolia* include cooling blood to stop bleeding, promoting blood circulation, and removing blood stasis. It is also used for blood heat hemoptysis, vomiting blood, epistaxis, blood in urine, blood in stool, disintegration and leakage, menstruation, postpartum stasis and abdominal pain, falling injury, rheumatism, and other diseases. Owing to the recent discovery of chemical components such as quinones, cyclohexeptides, and polysaccharides in the Indian madder, its pharmacological effects are gradually expanding and now include hemostasis and stasis removal, antioxidation, anti-inflammation, antitumor, immune regulation, and neuroprotective activities. *Rubia cordifolia* can also be used to inhibit macrophage inflammation and protect against kidney oxidative damage [8, 9]. Therefore, the rat model of myocardial ischemia-reperfusion was established in this study, and then, the rats were treated with *Rubia cordifolia* to explore the therapeutic effect of *Rubia cordifolia* on ischemia-reperfusion injury.

## 2. Materials and Methods

### 2.1. Modeling and Treatment of Experimental Animals

*Rubia cordifolia* root material was purchased from the Wandong Bridge Chinese herbal medicine market in Nanming District, Guiyang City, Guizhou Province, and was positively identified by Associate Professor Long Qingde of Guizhou University of Traditional Chinese Medicine. The root material was boiled twice, and the decoctions from the two boilings were combined. The combined decoctions were filtered and concentrated to 1 g/mL, which is equivalent to raw material, and stored at 4°C for later use. This experiment has been approved by the Ethics Committee of Qionghai People's Hospital (Project No.: QH-20201120-0012).

Thirty-two healthy adult SD rats were randomly divided into the sham group (sham), the model group (model), the madder group (madder), and the diltiazem group (Dil). Seven days before surgery, rats in the model group and the sham group were injected with 1 mL normal saline every day, rats in the madder group were injected with madder extract (0.5 mg/kg) every day, and rats in the Dil group were injected with diltiazem (0.2 mg/kg) every day. I/R modeling was performed in the model group, the madder group, and the Dil group.

For the performance of the myocardial ischemia-reperfusion model [10], rats were anesthetized with pentobarbital sodium and immobilized on the operating table. Endotracheal intubation was performed on the rats, and a positive pressure ventilator was implanted to assist respiration. Left 3–4 rib space thoracotomy was done to break the pericardium to expose the heart. The left anterior descending coronary artery was ligated using a surgical lead. After ligation of the anterior descending branch for 30 min, the ligation line was relaxed, and reperfusion was performed for 1 h. The sham operation group was not subjected to ligation of the anterior descending branch, but the other steps were the same. Arterial flow and myocardial systolic rate were measured after operation.

### 2.2. Coronary Blood Flow Detection

The right atrial outflow was measured after reperfusion, and the coronary blood flow (CF) rate was observed at 5 min, 10 min, 20 min, and 30 min after reperfusion. The flow rate of stabilizer > 8 mL/min of the heart rate was selected. Coronary outflow (CE) was collected at the end of the test. During the experiment, myocardial contractility was recorded by the frog heart clip.

### 2.3. Triphenyl Tetrazolium Chloride (TTC) Staining Was Used to Determine the Area of Myocardial Infarction

After the mice were sacrificed, the heart tissue was taken out quickly, before the atria and right ventricle were cut out, and stored in 4°C PBS solution. The heart tissue was then frozen at −20°C for 30 min. The heart tissue was taken out and sectioned, before the sections were placed in 2% TTC solution and stained in darkness at 37°C for 30 min. After staining, the heart sections were fixed with neutral formaldehyde fixative for 24 h. The heart section was photographed, and the risk area, infarct area, and total area were analyzed by ImageJ software.

### 2.4. The Related Factors Were Determined by ELISA

The TNF-*α* ELISA kit (Solarbio Life Science, Beijing) was used to detect TNF-*α*. The collected rat blood was placed at room temperature for 2 h and centrifuged at 1000 × g for 20 min before the supernatant was taken. After that, serum TNF-*α* concentration was detected by the TNF-*α* kit, and the detection steps were carried out according to the instructions. IL-1*β*, IL-6, Caspase-3, Caspase-9, LC3-II, ULK1, creatine kinase (CK), LDH, and ET-1 were detected using corresponding kits, and the operation was the same as above.

### 2.5. Determination of Functional Markers of Endothelial Cells

Serum NO content was determined by nitrate reductase. The nitric acid serum was taken from the −80°C refrigerator and thawed gradually (−80°C to −20°C to 4°C room temperature at 37°C water bath). A volume of 0.1 mL double steam water was added to the blank pair, 0.1 mL 100 *μ*mol/L standard application solution was added to the standard tube, 0.1 mL test sample was added to the test tube, and 0.2 mL reagent 1 and 0.2 mL reagent 2 were added to each tube, respectively, before mixing on the shaker and incubating at 37°C for 60 min. A volume of 0.2 mL of reagent 3 and 0.1 mL of reagent 4 were added to each of the above tubes, and they were fully vortex-mixed for 30 s. The tubes were left standing at room temperature for 10 min before they were centrifuged at 3500 RPM for 10 min. The supernatant was taken for color rendering. From each of the tubes, 0.45 mL of the supernatant was taken, and 0.6 mL chromogenic agent was added (Beijing Baiolibo Technology Co., LTD, SNM195) before mixing well on the vibrator, leaving them at room temperature for 10 min. The absorbance of each tube was measured against distilled water blank at 550 nm wavelength and 0.5 cm light diameter. Concentration was calculated as follows:

(1) $$\begin{array}{c} \text{Concentration}\left(\mu \text{mol}/\text{L}\right)=\frac{\text{test tube absorbance}-\text{blank tube absorbance}}{\text{standard tube absorbance}-\text{blank tube absorbance}}\times 100\times \text{dilution ratio of sample}. \end{array}$$

NOS activity was determined by chemical colorimetry. The determination method was as shown in the instructions of the nitric oxide synthase test box. In summary, 0.2 mL of double steam water was added to the blank control, 0.1 mL double steam water and 0.1 mL test samples were added to the total NOS (TNOS) test tube, and 0.1 mL inhibitor and 0.1 mL test samples were added to the iNOS test tube. A volume of 0.2 mL of substrate buffer, 10 *μ*L of accelerant, and 0.1 mL of color-developing agent were added to the above tubes before they were thoroughly mixed on the vibrator and incubated at 37°C for 15 min. A transparent agent (0.1 mL) and a stop solution (2 mL) were added to each of the above tubes before the contents were mixed well. The absorbance of each tube was measured after zeroing with distilled water, at 530 nm wavelength and 1 cm light diameter. NOS activity (U/mL) = (absorbance of TNOS determination tube − absorbance of blank tube)/nanomolar extinction coefficient of chromosome x (total volume of reaction solution/amount of solution taken) × 1/(specific color light diameter × reaction time) × 0.001.

### 2.6. Myocardial Histology Was Examined by HE Staining

A myocardial tissue (0.1 g) of the infarct area of mice in each group was fixed in 4% paraformaldehyde for more than 2 h. The fixed myocardial tissue was embedded in paraffin and sectioned. The slices were placed overnight in an oven at 60°C, immersed in xylene transparent, then hydrated with a series of concentration gradients of ethanol, and immersed in tap water to ensure full hydration. The slices were dyed with Harris hematoxylin for 3–8 min, washed with tap water, differentiated with 1% alcohol hydrochloride for several seconds, rinsed with tap water, and placed in distilled water for 5 min. From the distilled water, the slices were immersed in 0.5% eosin dyeing VAT for 10 min, rinsed with tap water for several seconds, and passed through a series of concentration gradient ethanol dehydration, xylene transparent, and neutral resin sealing. The slices were then examined under a microscope.

### 2.7. Activation of NF-*κ*B Pathway-Related Factors Was Detected by WB

Cardiac cytoplasm and nucleoprotein were extracted using a cytoplasm/nucleoprotein extraction kit. Part of the rat myocardium was taken and rinsed with precooled PBS containing 1‰ PMSF before a cell lysis buffer was added and homogenized. After homogenization, the contents were transferred to an EP tube for ultrasound, placed on ice for 10 min, and mixed. The precipitate and supernatant were separated by centrifugation at 500 × g at 4°C for 7 min. The supernatant was collected as cytoplasmic protein by centrifugation at 2000 × g at 4°C for 15 min and stored at −80°C for later use. The supernatant was simply blown, placed on ice for 2 min, and centrifuged at 500 × g at 4°C for 7 min before discarding the supernatant, adding 150 *μ*L nude storage buffer, and turning it upside-down several times to remove the precipitate. A volume of 1/10 of nude lysis buffer was added before blowing and shaking it at 900–1200 RPM at 4°C for 15 min and centrifuging at 20000 × g at 4°C for 5 min. The supernatant was taken and stored at −80°C. Proteins were isolated by SDS-PAGE (polyacrylamide) gel electrophoresis. A mass of 20 *μ*g of protein sample was transferred to nitrocellulose film at 100 V for 1 h and sealed at 37°C for 1 h before a primary antibody was added and incubation at 4°C overnight. TBST was washed for 3 × 15 min before a secondary antibody (1 : 1000/1 : 2000) was added, for 1 h at room temperature. TBST was washed again for 3 × 15 min. Western blotting was used to observe, and image analysis was used to determine the absorbance (*A*) value of each band for quantitative analysis.

### 2.8. Statistical Analysis

GraphPad Prism 8.0 (GraphPad Software, Inc, La Jolla, CA, USA) was used for statistical analysis. All results were expressed as the mean ± SD. WB result strips and optical density values were analyzed by the ImageJ software processing system. Differences between the experimental groups were assessed by the *t*-test or one-way analysis of variance and significance *T*-test (LSD). *P* < 0.05 was considered statistically significant.

## 3. Results

### 3.1. Treatment Can Effectively Reduce Myocardial Injury and Restore CF and Myocardial Contraction Rate

**Figure 1:**
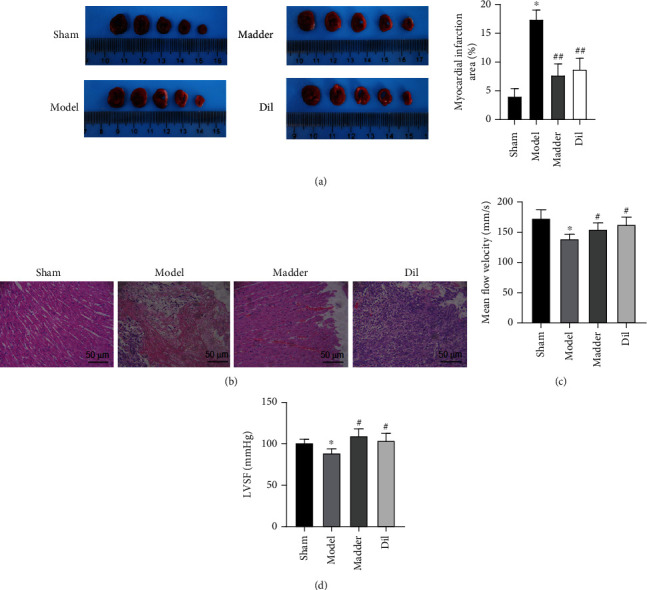
Effect of madder treatment on myocardial infarction and myocardial injury in rats: (a) TTC staining was used to determine the size of myocardial infarction in rats; (b) HE staining was used to detect the integrity of the myocardial structure and the degree of the lesion; (c) detection of coronary artery flow velocity in rats; (d) detection of systolic myocardial rate in rats. ^∗^*P* < 0.05, compared with the sham group; ^#^*P* < 0.05, compared with the model group.

The myocardial infarction area of mice can be quantified by TTC staining. As shown in Figure 1(a), the myocardial infarction area of mice in the model group was significantly larger than that in the sham group and was significantly reduced after treatment in the Dil group and the madder group. HE staining was used to examine the integrity of the myocardial structure and the degree of lesion. As shown in Figure 1(b), in the sham operation group, the myocardial membrane was intact, the myofibrils were balanced, and the adjacent myofibrils were continuous. In the model group, the heart was ischemic due to coronary ligation, resulting in a large number of inflammatory cells, swelling and deformation of myocardial cells, myocardial necrosis, and disappearance of transverse striations. The myocardial structure of the madder group and the Dil group was more intact than that of the sham operation group. Myocardial ischemia-reperfusion caused myocardial microvascular tissue damage and blocked coronary vessels, resulting in a significant decline in isolated coronary artery blood flow, as shown in Figure 1(c). CF in the model group decreased rapidly, and madder and Dil intervention significantly improved the declining trend. As shown in Figure 1(d), the left ventricular systolic function in the model group was significantly lower than that in the sham group, whereas madder and Dil intervention effectively restored myocardial contractility.

### 3.2. Madder Treatment Can Effectively Inhibit Inflammation and Protect Myocardial Cells from Excessive Autophagy and Apoptosis

**Figure 2:**
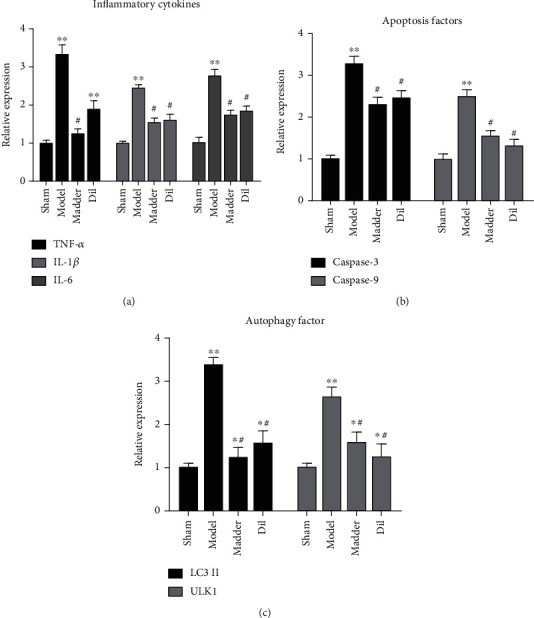
Effect of madder treatment on inflammatory response, autophagy, and apoptosis of myocardial cells: (a) the expressions of inflammatory cytokines TNF-*α*, IL-1*β*, and IL-6 were detected by ELISA assay; (b) the expression changes of apoptosis for factors Caspase-3 and Caspase-9 were detected by ELISA assay; (c) ELISA assay was used to detect the expression changes of autophagy factors LC3-II and ULKI. ^∗^*P* < 0.05 and^∗∗^*P* < 0.01, compared with the sham group; ^#^*P* < 0.05, compared with the model group.

Inflammatory factors are important causes of reperfusion injury. The expressions of TNF-*α*, IL-1*β*, and IL-6 inflammatory factors in the model group and the Dil group were significantly increased, whereas the levels of inflammatory factors in the madder group and the Dil group were significantly decreased (Figure 2(a)). Apoptosis is also an important process in I/R injury. The expression levels of apoptosis factors Caspase-3 and Caspase-9 were significantly increased in the model group (Figure 2(b)). The expression levels of Caspase-3 and Caspase-9 were significantly more downregulated in the Dil and madder groups than in the model group. In most cases, autophagy helps cells survive better, while excessive autophagy leads to cell death. The autophagy factor LC3-II in the model group was significantly higher than that in the sham group, and the autophagy factor in the Dil and madder groups was significantly lower than that in the model group and significantly higher than that in the sham group (Figure 2(c)).

### 3.3. Madder Can Effectively Protect Endothelial Cells and Cardiomyocytes in the Treatment of Myocardial I/R Injury

**Figure 3:**
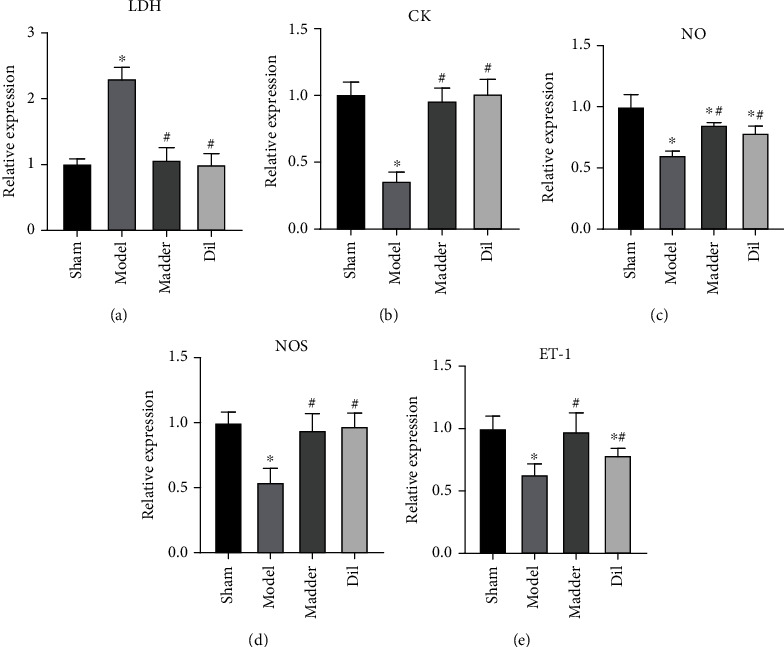
Effect of madder treatment on endothelial cell and myocardial cell function in rats: (a, b) ELISA assay was used to detect the expression of myocardial cell functional markers creatine kinase (CK) and lactate dehydrogenase (LDH); (c–e) ELISA assay was used to detect the expression of endothelial cell functional markers NO, NOS, and ET-1. ^∗^*P* < 0.05, compared with the sham group; ^#^*P* < 0.05, compared with the model group.

Endothelial dysfunction is an important marker and basis of I/R injury. LDH and CK are functional markers of cardiac myocytes, whereas NO, NOS, and ET-1 are functional markers of endothelial cells, which can reflect the integrity of cell function. LDH expression was significantly increased in the model group, while there was no significant change in the madder group and the Dil group (Figure 3(a)). CK in the model group decreased significantly, whereas there was no significant change in the Dil group and the madder group (Figure 3(b)). NO expression in the model group decreased significantly, whereas NO expression in the madder group and the Dil group showed NO significant change (Figure 3(c)). NOS expression was significantly decreased in the model group, but no significant change was observed in the madder group and the Dil group (Figure 3(d)). The level of ET-1 in the model group was significantly decreased whereas that in the madder group was not significantly changed, and that in the Dil group was significantly decreased (Figure 3(e)).

### 3.4. Madder Can Effectively Inhibit the Activation of NF-*κ*B Pathway

**Figure 4:**
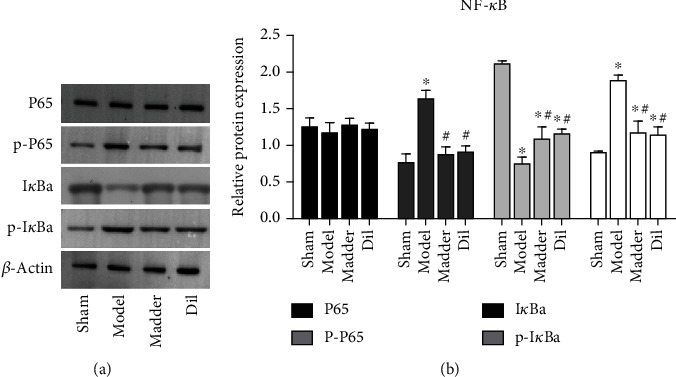
Effect of madder treatment on NF-*κ*B pathway. The expression levels of NF-*κ*B pathway-related proteins P65, P-P65, I*κ*Ba, and P-I*κ*Ba were detected by WB assay. ^∗^*P* < 0.05, compared with the sham group; ^#^*P* < 0.05, compared with the model group.

Activation of the NF-*κ*B pathway can activate inflammatory response [11, 12], and the activation of this pathway can be determined by detecting the levels of P65, P-P65, I*κ*Ba, and P-I*κ*Ba in the nucleus and in the cell. The levels of P-P65 and P-I*κ*Ba in the model group were significantly increased, and the protein expression of P-P65 and P-I*κ*Ba in the madder and Dil groups was significantly more decreased than that of the model group (Figure 4(a)). The NF-*κ*B pathway was activated in the model group whereas the activity of the NF-*κ*B pathway was inhibited after madder treatment.

## 4. Discussion

The main mechanisms of I/R injury include oxidative stress injury, intracellular calcium overload, drastic changes in physiological pH, inflammatory response, and the opening of the mitochondrial permeability transition pore. At the beginning of ischemia-reperfusion, there is a strong oxidative stress response that mediates a variety of mechanisms leading to myocardial cell damage and death. Clinically, however, antioxidant deficiency has been treated with mixed results, which may be partly due to the inability of antioxidants to enter cells. Specific antioxidants targeting mitochondria may be more effective [13]. When myocardial ischemia occurs, intracellular calcium overload begins. Calcium overload leads to mitochondrial dysfunction, and many enzymes are activated by calcium ions, prompting the excessive contraction of myocardial fibers and more. In addition, calcium overload can also promote the opening of mitochondrial permeability transition pores. In the clinical practice of myocardial ischemia-reperfusion injury, the use of calcium antagonists has not achieved ideal efficacy [14]. In recent years, continuous breakthroughs have been made in the study of unidirectional mitochondrial transporters, and these researches are expected to find specific inhibitors for myocardial reperfusion injury [15]. The mitochondrial membrane permeability conversion pore is the nonselective channel of the mitochondrial intima, and the opening of the intima leads to the depolarization and oxidative phosphorylation of the mitochondrial membrane, further leading to ATP loss and cell death [16]. During acute myocardial ischemia, intracellular pH can drop below 7.0; during reperfusion, physiological pH recovers rapidly due to the elution of lactic acid, resulting in excessive contracture of cardiomyocytes and inducing cardiomyocyte death. Therefore, during myocardial reperfusion, drugs can be used to inhibit Na+–H+ exchange or slow down the process of myocardial reperfusion to avoid fatal myocardial reperfusion injury [17]. In this study, we treated rats with 0.5 mg/kg of madder extract intravenously; in similar previous studies, dosages of 100–400 mg/kg (orally) of madder extract were effective and nontoxic [18]. Although the dose used in this study was much less, the results were similar to those of previous studies in that they showed a significant treatment effect of madder and no drug toxicity.

Microcirculation injury is one of the most important pathological mechanisms of I/R injury [19, 20]. Endothelial cells are the basis of heart microcirculation. I/R injury can result in endothelial cell injury, edema, hyperemia, and inflammatory cell infiltration. Moreover, endothelial cell dysfunction can result in basic NO synthesis decreased and vascular tension increased. The formation of superoxide anion increased, leading to oxidative stress injury and aggravating I/R injury. The autophagy of endothelial cells can improve cell activity and survival ability. Autophagy reactivation of vascular endothelial cells is rare in normal physiological processes and occurs in the pathological adaptive responses of vascular endothelial cells [21]. Experiments have confirmed that I/R injury can promote the activation of nuclear factor KB-P65 and regulate autophagy through Beclin1, ultimately aggravating endothelial cell injury [22]. In addition, autophagy is also involved in regulating other important functions of endothelial cells, such as NO production and release as well as angiogenesis and hemostasis/thrombosis [23].

Many methods have been used to prevent and treat ischemia-reperfusion injury. Ischemic preconditioning can effectively reduce the size of myocardial infarction by inducing short-term, nonlethal ischemia-reperfusion multiple times before the occurrence of long-term ischemia to activate the body's protective mechanism [24]. On this basis, ischemia tolerance is induced by bioactive substances. This method is feasible for clinical application; it is also predictable, controllable, safe, and easy to conduct. Studies have shown that treatment with tanshinone IIA was found to have an antimyocardial I/R injury effect [25]. In addition, relevant studies have shown that ORF scavengers can significantly improve systolic myocardial rate, cardiac function, reperfusion arrhythmias, and temporary cardiac arrest [26]. Clinical studies have shown that allopurinol can reduce the peak of troponin in patients with acute ST-segment elevation myocardial infarction after PCI. Additionally, the incidence of adverse cardiac events decreased by 13% compared with the control group in the follow-up one month later [27].

## 5. Conclusion

In conclusion, madder can effectively reduce the size of myocardial infarction; inhibit inflammation, autophagy, and apoptosis; and protect the structural and functional integrity of myocardial cells and endothelial cells in the treatment of ischemia-reperfusion injury. However, the mechanism by which madder regulates NF-*κ*B activity is still unclear. Further experiments should be designed to explore the targets of madder regulating NF-*κ*B activity.

## Data Availability

The datasets used and/or analyzed during the current study are available from the corresponding author upon reasonable request.

## References

[1] Frank A., Bonney M., Bonney S., Weitzel L., Koeppen M., Eckle T. (2012). Myocardial ischemia reperfusion injury: from basic science to clinical bedside. *Seminars in Cardiothoracic and Vascular Anesthesia*.

[2] Li D., Lu N., Han J. (2018). Eriodictyol attenuates myocardial ischemia-reperfusion injury through the activation of JAK2. *Frontiers in Pharmacology*.

[3] Yellon D. M., Hausenloy D. J. (2007). Myocardial reperfusion injury. *The New England Journal of Medicine*.

[4] Singhal A. K., Symons J. D., Boudina S., Jaishy B., Shiu Y. T. (2010). Role of endothelial cells in myocardial ischemia-reperfusion injury. *Vascular Disease Prevention*.

[5] Lopez-Neblina F., Toledo A. H., Toledo-Pereyra L. H. (2005). Molecular biology of apoptosis in ischemia and reperfusion. *Journal of Investigative Surgery*.

[6] Ling Q., Yu X., Wang T., Wang S. G., Ye Z. Q., Liu J. H. (2017). Roles of the exogenous H_2_S-mediated SR-A signaling pathway in renal ischemia/reperfusion injury in regulating endoplasmic reticulum stress-induced autophagy in a rat model. *Cellular Physiology and Biochemistry*.

[7] Zhu J., Yao K., Wang Q. (2016). Ischemic postconditioning-regulated miR-499 protects the rat heart against ischemia/reperfusion injury by inhibiting apoptosis through PDCD4. *Cellular Physiology and Biochemistry*.

[8] Lodi S., Sharma V., Kansal L. (2011). The protective effect of Rubia cordifolia against lead nitrate-induced immune response impairment and kidney oxidative damage. *Indian journal of pharmacology*.

[9] Zhu Z. G., Jin H., Yu P. J., Tian Y. X., Zhang J. J., Wu S. G. (2013). Mollugin inhibits the inflammatory response in lipopolysaccharide-stimulated RAW264.7 macrophages by blocking the Janus kinase-signal transducers and activators of transcription signaling pathway. *Biological & Pharmaceutical Bulletin*.

[10] Liu X. M., Yang Z. M., Liu X. K. (2017). Fas/FasL induces myocardial cell apoptosis in myocardial ischemia-reperfusion rat model. *European Review for Medical and Pharmacological Sciences*.

[11] Morita H., Nishino H., Nakajima Y. (2015). Oxomollugin, a potential inhibitor of lipopolysaccharide-induced nitric oxide production including nuclear factor kappa B signals. *Journal of Natural Medicines*.

[12] Chakrabortee S., Liu Y., Zhang L. (2012). Macromolecular and small-molecule modulation of intracellular A*β*42 aggregation and associated toxicity. *The Biochemical Journal*.

[13] Smith R. A., Hartley R. C., Murphy M. P. (2011). Mitochondria-targeted small molecule therapeutics and probes. *Antioxidants & Redox Signaling*.

[14] Fröhlich G. M., Meier P., White S. K., Yellon D. M., Hausenloy D. J. (2013). Myocardial reperfusion injury: looking beyond primary PCI. *European Heart Journal*.

[15] De Stefani D., Raffaello A., Teardo E., Szabò I., Rizzuto R. (2011). A forty-kilodalton protein of the inner membrane is the mitochondrial calcium uniporter. *Nature*.

[16] Heusch G., Boengler K., Schulz R. (2010). Inhibition of mitochondrial permeability transition pore opening: the Holy Grail of cardioprotection. *Basic Research in Cardiology*.

[17] Sanada S., Komuro I., Kitakaze M. (2011). Pathophysiology of myocardial reperfusion injury: preconditioning, postconditioning, and translational aspects of protective measures. *American Journal of Physiology Heart and Circulatory Physiology*.

[18] Chandrashekar B. S., Prabhakara S., Mohan T. (2018). Characterization of Rubia cordifolia L. root extract and its evaluation of cardioprotective effect in Wistar rat model. *Indian journal of pharmacology*.

[19] Yan L., Vatner D. E., Kim S. J. (2005). Autophagy in chronically ischemic myocardium. *Proceedings of the National Academy of Sciences of the United States of America*.

[20] Jaffe R., Dick A., Strauss B. H. (2010). Prevention and treatment of microvascular obstruction-related myocardial injury and coronary no-reflow following percutaneous coronary intervention: a systematic approach. *JACC: Cardiovascular Interventions*.

[21] Lavandero S., Chiong M., Rothermel B. A., Hill J. A. (2015). Autophagy in cardiovascular biology. *The Journal of Clinical Investigation*.

[22] Zeng M., Wei X., Wu Z. (2014). Reactive oxygen species contribute to simulated ischemia/reperfusion-induced autophagic cell death in human umbilical vein endothelial cells. *Medical Science Monitor*.

[23] Jiang F. (2016). Autophagy in vascular endothelial cells. *Clinical and Experimental Pharmacology & Physiology*.

[24] Narayanan S. V., Dave K. R., Perez-Pinzon M. A. (2013). Ischemic preconditioning and clinical scenarios. *Current Opinion in Neurology*.

[25] Yuan X., Jing S., Wu L., Chen L., Fang J. (2014). Pharmacological postconditioning with tanshinone IIA attenuates myocardial ischemia-reperfusion injury in rats by activating the phosphatidylinositol 3-kinase pathway. *Experimental and Therapeutic Medicine*.

[26] Kanaan G. N., Harper M. E. (2017). Cellular redox dysfunction in the development of cardiovascular diseases. *Biochimica et Biophysica Acta (BBA)-General Subjects*.

[27] Rentoukas E., Tsarouhas K., Tsitsimpikou C., Lazaros G., Deftereos S., Vavetsi S. (2010). The prognostic impact of allopurinol in patients with acute myocardial infarction undergoing primary percutaneous coronary intervention. *International Journal of Cardiology*.

